# Mitochondrial genome editing of *WA352* via mitoTALENs restore fertility in cytoplasmic male sterile rice

**DOI:** 10.1111/pbi.14315

**Published:** 2024-02-26

**Authors:** Jiawei Zhou, Liyun Nie, Shuo Zhang, Hailiang Mao, Shin-ichi Arimura, Shuangxia Jin, Zhiqiang Wu

**Affiliations:** ^1^ College of Plant Science and Technology Huazhong Agricultural University Wuhan China; ^2^ Shenzhen Branch, Guangdong Laboratory of Lingnan Modern Agriculture, Key Laboratory of Synthetic Biology, Laboratory of the Ministry of Agriculture and Rural Affairs Agricultural Genomics Institute at Shenzhen, Chinese Academy of Agricultural Sciences Shenzhen China; ^3^ National Key Laboratory of Crop Genetic Improvement Huazhong Agricultural University Wuhan China; ^4^ Graduate School of Agricultural and Life Science University of Tokyo Tokyo Japan

**Keywords:** mitochondrial genome, *WA352*, mitoTALENs, cytoplasmic male sterile, rice

Cytoplasmic male sterility (CMS) is the inability of plants to produce functional pollen due to nucleo‐cytoplasmic interaction (Wang *et al*., 2021). The CMS line is widely used in the three‐line (CMS, maintainer and restorer) system to produce hybrid seeds with heterosis (Chen and Liu, 2014). Rice is one of the three major food crops in the world, and CMS‐based hybrid rice technology has greatly improved the yield, among which the wild‐abortive type CMS (CMS‐WA) line is the most widely used (Tang *et al*., 2014).

*WA352*, also named *WA352c*, is a CMS‐associated gene of CMS‐WA rice. Luo *et al*. (2013) transferred *WA352* with mitochondrial transit signal into ZH11, indirectly proving that *WA352* can cause CMS. *orf352* in CMS‐RT102 rice, which has only five nucleotide differences from *WA352* and thus lead to four amino acid substitutions (Tang *et al*., 2017). Omukai *et al*. (2021) deleted *orf352* via TALEN with mitochondrial localization signal (MLS; mitoTALENs), but the mutants only restored pollen viability and did not restore seed setting. In this study, we utilized mitoTALENs to directly edit *WA352* in CMS‐WA type rice to study the pollen viability and seed setting rate of edited offspring.

*WA352* is a chimeric gene mainly composed of *orf284* (100% nucleotide identities), unique region, *orf224* (81%) and *orf288* (97%) (Luo *et al*., 2013, Figure 1a). We designed a target in the unique region and constructed the *WA352*::mitoTALEN vector (Figure 1a, Figure S1). The vector was transferred into an *indica* rice CMS‐WA line Jin23A via *Agrobacterium*‐mediated transformation, and we obtained 17 independent T_0_ plants.

**Figure 1:**
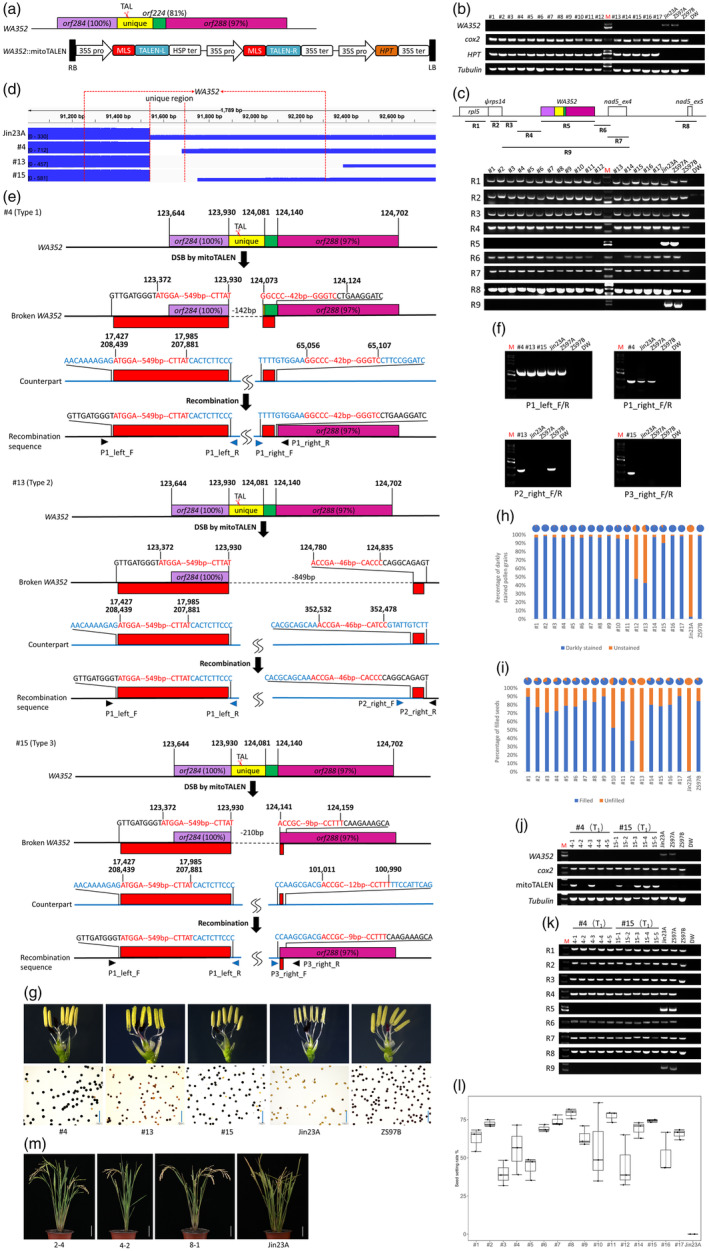
*WA352* triggers cytoplasmic male sterility in CMS‐WA rice. (a) Gene structure of *WA352* and the T‐DNA region of *WA352*::mitoTALEN vector. TAL is the target of *WA352*. (b) PCR analysis of *HPT* and *WA352* in T_0_ plants. M, DNA marker; Jin23A and ZS97A include *WA352*; ZS97B does not include *WA352*; DW, ddH_2_O; *cox2*, mitochondrial gene control; *Tubulin*, nuclear gene control. (c) PCR analysis of *WA352* and its vicinity in T_0_ plants. (d) PacBio HiFi sequencing coverage of *WA352* and its vicinity in T_0_ plants. (e) Homologous recombination repair mechanism of DSB generated by mitoTALENs. Counterpart, the repair template; the numbers above the bases indicate their position on Jin23A mitochondrial genome. (f) PCR analysis of new recombination sequences in T_0_ plants. (g–i) Disruption of *WA352* restores pollen viability and seed setting of T_0_ plants. Bars, 200 μm. (j) PCR analysis of *WA352* and mitoTALEN in T_1_ plants. (k) PCR analysis of *WA352* and its vicinity in T_1_ plants. (l) Seed setting rate of T_1_ plants. (m) The growth state of T_1_ plants. Bars, 3 cm.

First, we amplified an introduced hygromycin resistance gene (*HPT*) and the coding region of *WA352* by PCR. The results showed that all T_0_ plants were vector positive and all achieved deletion of *WA352* (Figure 1b). Previous study has shown that mitoTALENs can lead to the deletion of target and its vicinity (Omukai *et al*., 2021). To analyse the extent of the deletion, we conducted PCR detection on eight regions (R1‐8) around *WA352* (Figure 1c). Agarose gel electrophoresis results showed that #12 and #13 achieved larger deletion around *WA352* than the other T_0_ plants. We conducted PacBio HiFi sequencing on Jin23A, #4, #13 and #15. We assembled the mitochondrial genome of Jin23A, mapped the reads of #4, #13 and #15 to the contig where *WA352* related, respectively, and visualized the results with Integrative Genomics Viewer (IGV) (Figure 1d). Compared with Jin23A, we found deletions of 142 bp, 849 bp and 210 bp around the *WA352* target in the #4, #13 and #15 (Figure 1d, Figure S2).

Kazama *et al*. (2019) show that mitoTALEN‐introduced DNA double‐strand breaks (DSB) are repaired by ectopic homologous recombination. We preliminarily confirmed this conclusion by detecting region 9 in Figure 1c. To analyse the repair mechanism of DSB, we assembled the mitochondrial genomes of #4, #13 and #15. Through comparison and analysis with Jin23A, we found that homologous recombination events occurred at the left‐side and right‐side free ends, respectively. The same recombination event mediated by 559 bp repeats occurred at the left‐side free end of #4, #13 and #15, while three different types of recombination events occurred at the right‐side free end of #4, #13 and #15, respectively (Figure 1e). For example, a homologous recombination mediated by 52 bp repeats occurred at the right‐side free end of #4 (type 1), a 19 bp repeat at the right‐side free end of #13 (type 2), and a 56 bp repeat at the right‐side free end of #15 (type 3) (Figure 1e). Sequence‐specific primers were used to detect the results of recombination at the left‐side and right‐side ends by PCR and Sanger sequencing (Figure 1f, Figure S3). We also predicted several new genes at 3′‐recombination sites via Open Reading Frame Finder (ORFfinder) (Table S3). These results illustrated that the deletion of *WA352* mediated by mitoTALENs is produced by causing a DSB first and then homologous recombination with the same or similar sequence as the template at the free end.

Next, we conducted statistical analysis on pollen viability and seed setting rate of T_0_ plants. Compared with Jin23A, the mutants showed different degree of restoration of pollen viability and seed setting (Figure 1g–i, Tables S1 and S2). To analyse the genetic stability, we obtained T_1_ plants derived from the self‐pollination of T_0_ plants. We amplified *WA352* of T_1_ plants by PCR, and agarose gel electrophoresis results showed that no plant could amplify *WA352* (Figure 1j). Meanwhile, we conducted PCR analysis on nine regions around *WA352* in T_1_ plants obtained from self‐pollination of #4 and #15, which all achieved the deletion of *WA352* (Figure 1k). Finally, the seed setting rate of #1 to #17 T_1_ plants were statistically analysed (Figure 1l). These results indicated recovery of pollen viability and seed setting in mutant offspring, confirming the genetic stability of mitoTALENs‐mediated gene editing offspring.

In summary, we successfully utilized mitoTALENs to edit rice CMS‐associated gene *WA352* and obtained T_0_ plants that restored pollen viability and seed setting, which directly confirmed that *WA352* was the cause of CMS. The differences in the mutant phenotypes caused by the editing results of *WA352* and *orf352* may be related to the differences in the nucleus of the receptor materials where they are located, which requires further analysis. In addition, we found that the *WA352*‐disrupted mitochondrial genome could be stably inherited in the next generation, even lacking mitoTALENs expression cassette. Our results are consistent with those of previous studies that mitoTALEN‐mediated DSBs are repaired by ectopic homologous recombination, which can lead to the deletion of hundreds of bases around the target. Our results confirm that mitoTALENs can effectively realize the functional study of plant mitochondrial genes, which will lay a good research foundation for crop organelle gene editing breeding research in future.

## Conflict of interest

The authors declare no conflict of interest.

## Author contributions

Z.W. designed the study. S.J., S.A. and H.M. provided technical assistance. J.Z. performed the experiments and prepared the manuscript. L.N. and S.Z. provided assistance with data analysis.

## Supporting information


**Appendix S1** Methods.

**Figure S1** Nucleotide sequences of the *WA352* coding region.
**Figure S2** Three types of deletion results of *WA352* and its surrounding sequences in #4, #13, and #15 T_0_ plants induced by mitoTALENs.
**Figure S3** Verification of recombination sequences in #4, #13, and #15 T_0_ plants.

**Table S1** Statistics on the percentage of darkly stained and unstained pollen in T_0_ plants.

**Table S2** Statistics on the percentage of filled and unfilled spikelets of T_0_ plants.

**Table S3** Chimeric loci generated by recombination at the 3′‐site.

**Table S4** Primers used in this study.

## Data Availability

The data that supports the findings of this study are available in the supplementary material of this article.

## References

[pbi14315-bib-0001] Chen, L.T. and Liu, Y.G. (2014) Male sterility and fertility restoration in crops. Annu. Rev. Plant Biol. 65, 579–606. 24313845 10.1146/annurev-arplant-050213-040119

[pbi14315-bib-0002] Kazama, T. , Okuno, M. , Watari, Y. , Yanase, S. , Koizuka, C. , Tsuruta, Y. , Sugaya, H. *et al*. (2019) Curing cytoplasmic male sterility via TALEN‐mediated mitochondrial genome editing. Nat. Plants. 5, 722–730. 31285556 10.1038/s41477-019-0459-z

[pbi14315-bib-0003] Luo, D.P. , Xu, H. , Liu, Z.L. , Guo, J.X. , Li, H.Y. , Chen, L.T. , Fang, C. *et al*. (2013) A detrimental mitochondrial‐nuclear interaction causes cytoplasmic male sterility in rice. Nat. Genet. 45, 573–577. 23502780 10.1038/ng.2570

[pbi14315-bib-0004] Omukai, S. , Arimura, S.I. , Toriyama, K. and Kazama, T. (2021) Disruption of mitochondrial *open reading frame* 352 partially restores pollen development in cytoplasmic male sterile rice. Plant Physiol. 187, 236–246. 34015134 10.1093/plphys/kiab236 PMC8418389

[pbi14315-bib-0005] Tang, H.W. , Luo, D.P. , Zhou, D.G. , Zhang, Q.Y. , Tian, D.S. , Zheng, X.M. , Chen, L.T. *et al*. (2014) The rice restorer *Rf4* for wild‐abortive cytoplasmic male sterility encodes a mitochondrial‐localized PPR protein that functions in reduction of *WA352* transcripts. Mol. Plant 7, 1497–1500. 24728538 10.1093/mp/ssu047

[pbi14315-bib-0006] Tang, H.W. , Zheng, X.M. , Li, C.L. , Xie, X.R. , Chen, Y.L. , Chen, L.T. , Zhao, X.C. *et al*. (2017) Multi‐step formation, evolution, and functionalization of new cytoplasmic male sterility genes in the plant mitochondrial genomes. Cell Res. 27, 130–146. 27725674 10.1038/cr.2016.115 PMC5223224

[pbi14315-bib-0007] Wang, C.D. , Lezhneva, L. , Arnal, N. , Quadrado, M. and Mireau, H. (2021) The radish Ogura fertility restorer impedes translation elongation along its cognate CMS‐causing mRNA. Proc. Natl. Acad. Sci. USA 118, e2105274118. 34433671 10.1073/pnas.2105274118 PMC8536381

