# Supplementary material for: Mitochondrial genome editing of WA352 via mitoTALENs restore fertility in cytoplasmic male sterile rice
Source: Plant Biotechnol J. 2024 Feb 26;22(7):1960–2. doi: 10.1111/pbi.14315 (PMC11182578; doi:10.1111/pbi.14315)
Supplement: Supplementary file 1 — Appendix S1 Methods. [file PBI-22-1960-s004.docx]

Supplemental Methods for

**Mitochondrial genome editing of *WA352* via mitoTALENs restore fertility in** **cytoplasmic male sterile rice**

**Methods**

**Vector construction of mitoTALEN and rice transformation**

Selection of target sequences and construction of mitoTALEN vector were as described previously (Kazama et al., 2019). DNA recognition motifs of the target sequences were assembled by Golden Gate cloning using the Platinum Gate TALEN kit into multisite entry vectors for the left and right TALEN ORFs (Sakuma et al., 2013). Then, these ORFs in the entry vectors were recombined into Gateway destination vector which is based on the binary vector pH7WG and contains CaMV 35S promoter, MLS, and Arabidopsis heat shock protein terminator (HSP18.2) via multisite LR reaction (Karimi et al., 2002). The wild type *indica* rice CMS-WA line Jin23A were used for plant transformation. mitoTALEN vector was introduced into *Agrobacterium tumefaciens* strain EHA105 and *Agrobacterium-*mediated transformation of embryogenic calli were performed by Biorun Biotechnology Company (Wuhan, China). Currently, we have obtained 17 T_0_ plants.

**DNA extraction and PCR**

Total DNA were extracted from green leaf blades via CTAB method. Briefly, about 0.1 g fresh rice leaves and steel balls in a 2 ml centrifuge tube were frozen in liquid nitrogen for 10 min, and then ground the samples in a grinder at 30 HZ, 60 s/time, twice; added 700 µl 2 × CTAB extraction buffer, took a 65℃ water bath for 40 min and gently inverted and mixed them every 10 min; added equal volume of nucleic acid extraction solution 24: 1 and shaked gently in a shaker for 20 min; centrifuged at 10000 rpm for 18 min and transferred the supernatants to clean 2 ml centrifuge tubes, respectively; added twice the volume of anhydrous ethanol and placed at -20℃ for 30 min to 1 hour; centrifuged at 12000 rpm for 10 min and then discard the waste liquid; washed DNA with 75% ethanol twice and centrifuged at 12000 rpm for 1 min to remove excess alcohol; put at room temperature for 30-40 min to remove excess alcohol; added 100 ul ddH_2_O to dissolve DNA precipitation. *HPT*, *WA352*, and the mitochondrial genome regions around *WA352* were determined by PCR using the primers in Table S4.

**PacBio HiFi sequencing, assembly, and long read coverage analysis**

Fresh green leaves of wild-type Jin23A and *WA352* deletion mutants were used for DNA extraction. PacBio HiFi sequencing services were provided by the Haorui Genomics Technology Co., Ltd. (Xi’an, China). SMRTbell target size libraries were constructed for sequencing according to PacBio’s standard protocol (Pacific Biosciences, CA). Sequencing was performed using the PacBio Sequel II platform in CCS mode, which generated a total of 56.9 Gb HiFi reads (19.6 Gb for wild-type Jin23A, 13.3 Gb for #4, 11.2 Gb for #13, and 12.8 Gb for #15). Highly accurate HiFi reads were used for *de* *novo* mitochondrial genome assembly using PMAT toolkit (<https://github.com/bichangwei/PMAT>). We used the “all” task mode provided by PMAT, which included broke the long HiFi reads into short reads (no more than 20 kb), and further assembled using Newbler v3.0 (Roche). Based on the assembled contig depth, mitochondria contigs were selected as extended seeds to capture all target mitochondrial contigs. After assembling, the simplified structure graphs were visualized with Bandage v0.8.1 (Wick et al., 2015).

The mitochondria genome of Jin23A was firstly assembled. The target *WA352* gene sequence was aligned to Jin23A mitochondria genome to extract the related contig (119 kb) using BLAST (Camacho et al., 2009). HiFi reads of mutants were mapped to *WA352* related contig (119 kb) using the map-hifi option in Minimap2 (Li 2018). The resulting SAM files were converted into BAM files and coordinately sorted using SAMtools v1.17 (Danecek et al., 2021). Bam files were visualized at the sequence level using IGV (Robinson et al., 2017). Based on the long read coverage analysis, we further confirmed that *WA352* gene sequences were successfully edited by mitoTALENs in T_0_ plants.

**Statistics of pollen viability and seed setting rate in T_0_ and T_1_ plants**

The growth of rice plants was photographed with camera (Nikon, Z 7ll). When the anther extends to two-thirds of the length of the glume, the pollen is considered mature. Used scissor and tweezer to remove the glume and retained the intact stamens, and captured the morphology of the stamens using a body style fluorescence microscope (Leica, M165FC). Placed the anther on a glass slide, use a dropper to drop 1-2 drops of 1% I_2_-KI Lugo’s solution, used tweezer to crush the anther to fully release the pollen grains. After staining for 2-3 min, the pollen grains were covered with a cover glass, and the pollen grain staining results were observed under a composite microscope (Olympus, BX53) and different fields of view were selected for photography. T_0_ plants were bagged and self-pollinated to obtain T_1_ plants, and the seed setting rate was calculated by counting the seeds on the filled and unfilled spikelets at maturity stage.

**References**

Camacho, C., Coulouris, G., Avagyan, V., Ma, N., Papadopoulos, J., Bealer, K. and Madden, T.L. (2009) BLAST+: architecture and applications. *BMC Bioinformatics*. **10**, 421.

Danecek, P., Bonfield, J. K., Liddle, J., Marshall, J., Ohan, V., Pollard, M.O., Whitwham, A. *et al*. (2021) Twelve years of SAMtools and BCFtools. *GigaScience*. **10**, giab008.

Karimi, M., Inzé, D., Depicker, A. (2002) GATEWAY^TM^ vectors for *Agrobacterium*-mediated plant transformation. *Trends Plant Sci*. **7**, 193–195.

Kazama, T., Okuno, M., Watari, Y., Yanase, S., Koizuka, C., Tsuruta, Y., Sugaya, H. *et al*. (2019) Curing cytoplasmic male sterility via TALEN-mediated mitochondrial genome editing. *Nat. Plants*. **5**, 722–730.

Li, H. (2018) Minimap2: pairwise alignment for nucleotide sequences. *Bioinformatics*. **34**, 3094-3100.

Robinson, J.T., Thorvaldsdóttir, H., Wenger, A.M., Zehir, A. and Mesirov, J.P. (2017) Variant Review with the Integrative Genomics Viewer. *Cancer Res*. **77**, e31–e34.

Sakuma, T., Ochiai, H., Kaneko, T., Mashimo, T., Tokumasu, D., Sakane, Y., Suzuki, K. *et al*. (2013) Repeating pattern of non-RVD variations in DNA-binding modules enhances TALEN activity. *Sci Rep*. **3**, 3379.

Wick, R.R., Schultz, M.B., Zobel, J. and Holt, K.E. (2015) Bandage: interactive visualization of *de novo* genome assemblies. *Bioinformatics*. **31**, 3350–3352.
