# Supplementary material for: Mitochondrial genome editing of WA352 via mitoTALENs restore fertility in cytoplasmic male sterile rice
Source: Plant Biotechnol J. 2024 Feb 26;22(7):1960–2. doi: 10.1111/pbi.14315 (PMC11182578; doi:10.1111/pbi.14315)
Supplement: Supplementary file 2 — Figure S1 Nucleotide sequences of the WA352 coding region. Figure S2 Three types of deletion results of WA352 and its surrounding sequences in #4, #13, and #15 T0 plants induced by mitoTALENs. Figure S3 Verification of recombination sequences in #4, #13, and #15 T0 plants. [file PBI-22-1960-s002.docx]

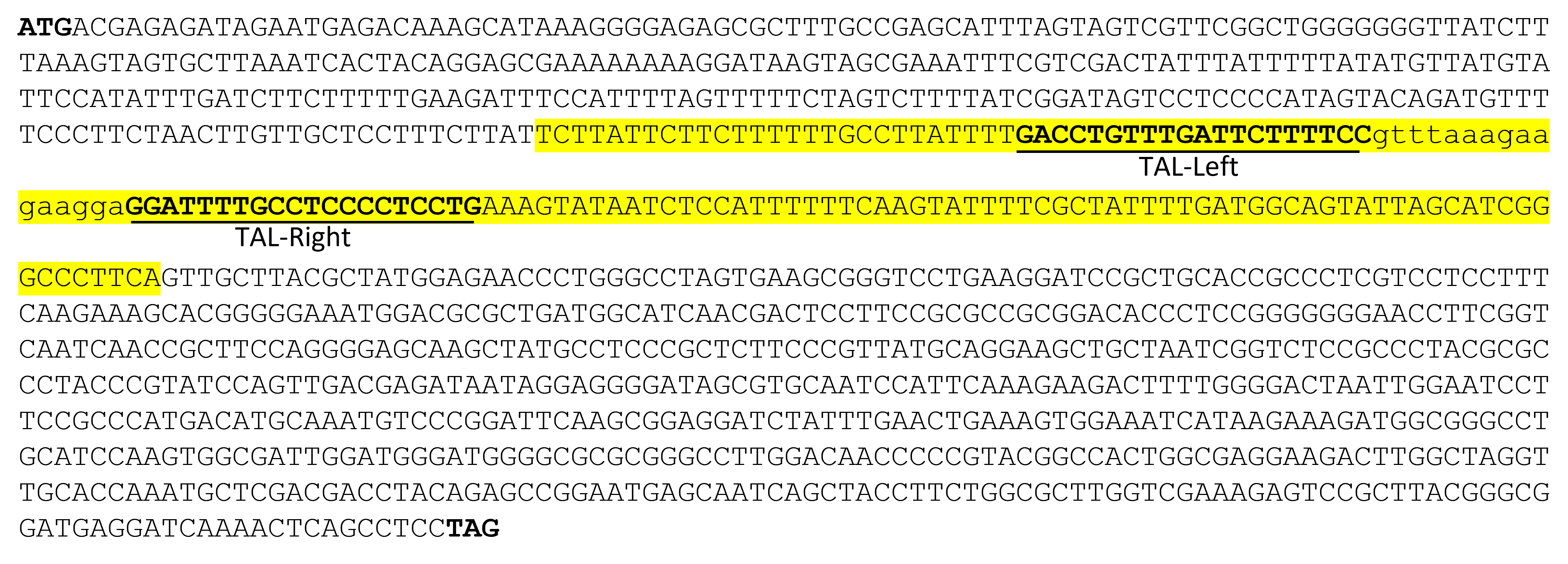


**Figure S1. Nucleotide sequences of the *WA352* coding region.** The sequence with a yellow background represents the unique region of *WA352*, and TAL-Left and TAL-Right are the binding sites of mitoTALENs on *WA352*.


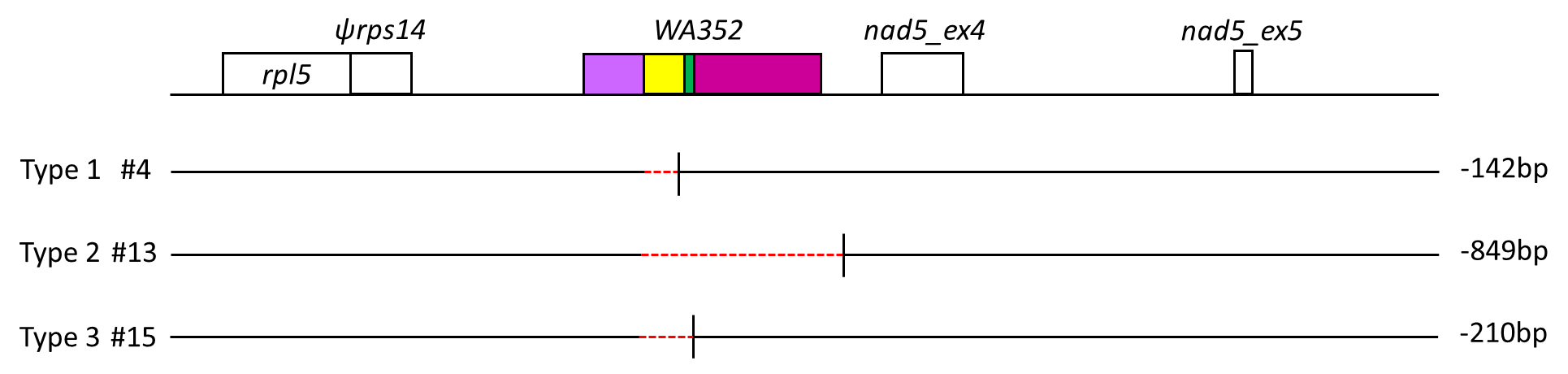


**Figure S2. Three types of deletion results of *WA352* and its surrounding sequences in #4, #13, and #15 T_0_ plants induced by mitoTALENs.** Red dashed lines indicate the locations of deleted regions in T_0_ plants.


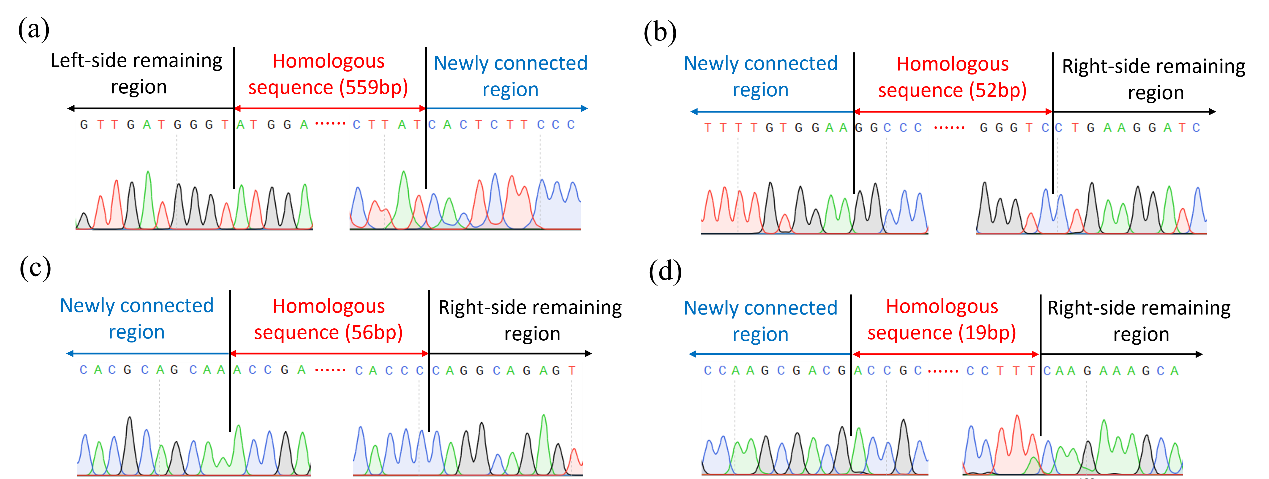


**Figure S3. Verification of recombination sequences in #4, #13, and #15 T0 plants.** Sequencing chromatogram obtained from Sanger sequencing of PCR products in Figure 1f. (a) Detection of recombination results occurring at the left-side free ends of #4, #13 and #15 T_0_ plants. (b), (c), and (d) indicate the detection of recombination results occurring at the right-side free ends of #4, #13, and #15 T_0_ plants, respectively.
