# Supplementary material for: Mitochondrial genome editing of WA352 via mitoTALENs restore fertility in cytoplasmic male sterile rice
Source: Plant Biotechnol J. 2024 Feb 26;22(7):1960–2. doi: 10.1111/pbi.14315 (PMC11182578; doi:10.1111/pbi.14315)
Supplement: Supplementary file 3 — Table S1 Statistics on the percentage of darkly stained and unstained pollen in T0 plants. [file PBI-22-1960-s005.pdf]

Table S1. Statistics on the percentage of darkly stained and unstained pollen in T_0 plants

T_0 plant	Darkly stained (%)	Unstained (%)	Total
#1	96.77	3.23	1
#2	98.52	1.48	1
#3	96.92	3.08	1
#4	96.67	3.33	1
#5	97.74	2.26	1
#6	96.59	3.41	1
#7	97.92	2.08	1
#8	96.67	3.33	1
#9	98.53	1.47	1
#10	95.62	4.38	1
#11	94.87	5.13	1
#12	47.67	52.33	1
#13	42.79	57.21	1
#14	97.28	2.72	1
#15	90.26	9.74	1
#16	98.90	1.10	1
#17	98.27	1.73	1
Jin23A	2.75	97.25	1
ZS97B	98.70	1.30	1
