# Supplementary material for: Mitochondrial genome editing of WA352 via mitoTALENs restore fertility in cytoplasmic male sterile rice
Source: Plant Biotechnol J. 2024 Feb 26;22(7):1960–2. doi: 10.1111/pbi.14315 (PMC11182578; doi:10.1111/pbi.14315)
Supplement: Supplementary file 4 — Table S2 Statistics on the percentage of filled and unfilled spikelets of T0 plants. [file PBI-22-1960-s003.pdf]

Table S2. Statistics on the percentage of filled and unfilled spikelets of T_0 plant

T_0 plant	Filled (%)	Unfilled (%)	Total
#1	89.66	10.34	1
#2	77.27	22.73	1
#3	70.83	29.17	1
#4	72.50	27.50	1
#5	78.95	21.05	1
#6	77.78	22.22	1
#7	85.19	14.81	1
#8	83.33	16.67	1
#9	90.00	10.00	1
#10	52.63	47.37	1
#11	84.21	15.79	1
#12	37.04	62.96	1
#13	0.00	100.00	1
#14	80.00	20.00	1
#15	78.26	21.74	1
#16	80.00	20.00	1
#17	90.32	9.68	1
Jin23A	99.00	1.00	1
ZS97B	84.48	15.52	1
