# Supplementary material for: Mitochondrial genome editing of WA352 via mitoTALENs restore fertility in cytoplasmic male sterile rice
Source: Plant Biotechnol J. 2024 Feb 26;22(7):1960–2. doi: 10.1111/pbi.14315 (PMC11182578; doi:10.1111/pbi.14315)
Supplement: Supplementary file 5 — Table S3 Chimeric loci generated by recombination at the 3′‐site. [file PBI-22-1960-s006.pdf]

Table S3. Chimeric loci generated by recombination at the 3'-site

Recombination type	New gene	Note
Type 1	*orf309*	Contains partial sequence of *WA352* (*orf288*)
Type 2	*orf90*	NA
Type 3	*orf84*	Contains partial sequence of *WA352* (*orf288*)
