# Supplementary material for: Mitochondrial genome editing of WA352 via mitoTALENs restore fertility in cytoplasmic male sterile rice
Source: Plant Biotechnol J. 2024 Feb 26;22(7):1960–2. doi: 10.1111/pbi.14315 (PMC11182578; doi:10.1111/pbi.14315)
Supplement: Supplementary file 6 — Table S4 Primers used in this study. [file PBI-22-1960-s001.pdf]

Table S4. Primers used in this study.

Primer name	Primer sequence (5' - 3')	Product size (bp)	Purpose
WA352_F	ATGACGAGAGATAGAATGAG	1056	PCR amplication of *WA352*
WA352_R	GGAGGCTGAGTTTTGATCCT		
cox2_F	CAGTTCCGATGAACAGTCAC	240	PCR amplication of *cox2*
cox2_R	TCTCGTTGTACCGAGATGGA		
HPT_F	GAGAGCCTGACCTATTGCAT	728	PCR amplication of *HPT*
HPT_R	TCGGCGAGTACTTCTACACA		
Tubulin_F	TGGTCGGATTCGCCCCGCTG	between 500 and 750	PCR amplication of *Tubulin*
Tubulin_R	TTACATGTCGTCAGCCTCCT		
R1_F	AGTACCAAAAGCTGCCTCTG	452	PCR amplication of R1 in Figure S2
R1_R	TTTCCCCCTCATCTTTTAGC		
R2_F	CACAAACGTAGATTGCTCGC	234	PCR amplication of R2 in Figure S2
R2_R	ATGCCCATCAAAGAACCTCG		
R3_F	CTTGGTAGCAACCACCAAAC	369	PCR amplication of R3 in Figure S2
R3_R	CCTCGGTGTCTCTATTTGCT		
R4_F	CAAATAGAGACACCGAGGCC	384	PCR amplication of R4 in Figure S2
R4_R	CGACTACTAAATGCTCGGCA		
R5_F	ATGACGAGAGATAGAATGAG	1056	PCR amplication of R5 in Figure S2
R5_R	GGAGGCTGAGTTTTGATCCT		
R6_F	AACTCAGCCTCCTAGACATG	277	PCR amplication of in R6 Figure S2
R6_R	AGTACGAAGGGGGAATTGG		
R7_F	CACTTGAATAAGCCGCCG	449	PCR amplication of R7 in Figure S2
R7_R	CTCTTCGCACAACAGATCC		
R8_F	ATTTAAGCAAGAGCAGCCGG	428	PCR amplication of R8 in Figure S2
R8_R	GGCAAGCTCCTACAGTTCTC		
R3_F	CTTGGTAGCAACCACCAAAC	2365	PCR amplication of R9 in Figure S2
R7_R	CTCTTCGCACAACAGATCC		
P1_left_F	CGGATCGGAGATGCTAACG	1146	PCR amplication of recombination sequence that were joined between the left-side free end and new connected region in #4
P1_left_R	GCTCGGATAGAGTCAAGATAGG		
P1_right_F	CTTCTGGTTCTGCCTTCTCCTA	454	PCR amplication of recombination sequence that were joined between the right-side free end and new connected region in #4
P1_right_R	CCTGGAAGCGGTTGATTGAC		
P2_right_F	CACTTCGGAGCACGGTCTTC	398	PCR amplication of recombination sequence that were joined between the right-side free end and new connected region in #13
P2_right_R	TGGAGCAGCAAACTCGGATTC		
P3_right_F	GGAGGCTGTATTGGCGAGAT	236	PCR amplication of recombination sequence that were joined between the right-side free end and new connected region in #15
P3_right_R	CCTGGAAGCGGTTGATTGAC		
